# MS2Planner: improved fragmentation spectra coverage in untargeted mass spectrometry by iterative optimized data acquisition

**DOI:** 10.1093/bioinformatics/btab279

**Published:** 2021-07-12

**Authors:** Zeyuan Zuo, Liu Cao, Louis-Félix Nothia, Hosein Mohimani

**Affiliations:** Computational Biology Department, Carnegie Mellon University, Pittsburgh, PA 15213, USA; Computational Biology Department, Carnegie Mellon University, Pittsburgh, PA 15213, USA; Skaggs School of Pharmacy and Pharmaceutical Sciences, University of California San Diego, La Jolla, CA 92093, USA; Computational Biology Department, Carnegie Mellon University, Pittsburgh, PA 15213, USA

## Abstract

**Motivation:**

Untargeted mass spectrometry experiments enable the profiling of metabolites in complex biological samples. The collected fragmentation spectra are the metabolite’s fingerprints that are used for molecule identification and discovery. Two main mass spectrometry strategies exist for the collection of fragmentation spectra: data-dependent acquisition (DDA) and data-independent acquisition (DIA). In the DIA strategy, all the metabolites ions in predefined mass-to-charge ratio ranges are co-isolated and co-fragmented, resulting in multiplexed fragmentation spectra that are challenging to annotate. In contrast, in the DDA strategy, fragmentation spectra are dynamically and specifically collected for the most abundant ions observed, causing redundancy and sub-optimal fragmentation spectra collection. Yet, DDA results in less multiplexed fragmentation spectra that can be readily annotated.

**Results:**

We introduce the MS2Planner workflow, an Iterative Optimized Data Acquisition strategy that optimizes the number of high-quality fragmentation spectra over multiple experimental acquisitions using topological sorting. Our results showed that MS2Planner increases the annotation rate by 38.6% and is 62.5% more sensitive and 9.4% more specific compared to DDA.

**Availability and implementation:**

MS2Planner code is available at https://github.com/mohimanilab/MS2Planner. The generation of the inclusion list from MS2Planner was performed with python scripts available at https://github.com/lfnothias/IODA_MS.

**Supplementary information:**

Supplementary data are available at *Bioinformatics* online.

## 1 Introduction

**Fig. 1.**
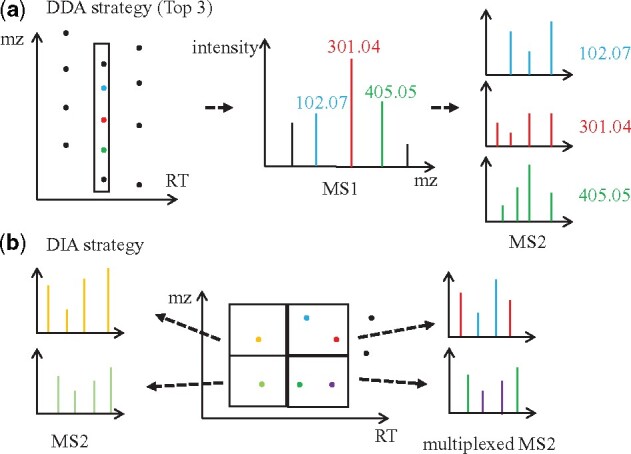
Comparison ofDDA and DIA strategy. (**a**) DDA strategy acquires a survey full-scan mass spectrum to select top *N* most abundant precursor ions for fragmentation spectra acquisition in the next several MS2 scans. (**b**) DIA fragments all the available precursors for MS2 scans in large predefined *m/z* and RT windows. The resulting MS2 are typically multiplexed and require further spectral deconvolution

Untargeted tandem mass spectrometry is widely used for analysis of proteins and metabolites in complex biological samples (Koelmel *et al.*, 2017; Schrimpe-Rutledge *et al.*, 2016; Wang *et al.*, 2019; Zha *et al.*, 2018). Data-dependent acquisition (DDA) is the most commonly used strategy to collect fragmentation mass spectra (MS2) (Hu *et al.*, 2016; Rudomin *et al.*, 2009). The fragmentation spectra are then identified by searching into spectral libraries or annotated with *in silico* tools (Cao *et al.*, 2019; Mohimani *et al.*, 2017, 2018). In the standard DDA strategy, a survey full-scan mass spectrum (MS1) is periodically acquired to inform on the selection of top *N* most abundant precursor ions for subsequent MS2 acquisition, where *N* is often between 3 and 10 precursor ions per cycle (Fig. 1a). While the sample complexity and the acquisition parameters influence the performance of DDA, many less abundant ions are left without any fragmentation spectra (Koelmel *et al.*, 2017; Rudomin *et al.*, 2009). Moreover, a portion of MS2 spectra collected in DDA have a low quality because they were collected at sub-optimal intensity levels. Repeating DDA on the same sample leads to the recollection of the most abundant ions and marginally improves the MS2 coverage (Table 1) (Hoopmann *et al.*, 2009; Kreimer *et al.*, 2016). One way to circumvent these disadvantages is by marking the precursor ions sampled in the previous run to exclude them from the next experiments in an iterative fashion (Hodge *et al.*, 2013; Koelmel *et al.*, 2017). Recently, dataset-dependent acquisition (DsDA) was introduced on quadrupole time-of-flight mass spectrometer to iteratively prioritize MS2 collection on the entire dataset in an automated fashion (Broeckling *et al.*, 2018). However, neither DDA nor DsDA optimizes the number of unique high-quality MS2 acquired for MS1 features.

**Table 1.** Comparison of different MS2 acquisition methods in untargeted tandem mass spectrometry experiments

	Sensitivity	Specificity	Coverage	Multiplexed spectra
DDA	Medium	Medium	Low	Infrequent
DIA	Low	Low	High	Frequent
IODA with MS2Planner	High	High	Medium	Infrequent

***Note*:** Sensitivity stands for the ratio of molecules present in the sample that can be identified by spectral library search. Specificity stands for the ratio of ions identified by spectral library search that correspond to a molecule in the sample. The coverage indicates proportion of ions with a fragmentation spectra collected. Note that high coverage is not equivalent to high sensitivity, since the spectra can be low quality/multiplexed, making it impossible to identify them by spectral library search. Note that due to the differential ionization response of each metabolites and instrument limit of detection, the sensitivity is fundamentally limited to a portion of molecules in these mass spectrometry experiments. Table is concluded from Wang *et al.* (2019), Broeckling *et al.* (2018), Schrimpe-Rutledge *et al.* (2016), Bilbao *et al.* (2015), Hu *et al.* (2016), Bauer *et al.* (2014), Vidova and Spacil (2017), Amodei *et al.* (2019).

Data-independent acquisition (DIA) systematically fragments all the available precursors for MS2 scans in large predefined *m/z* windows over time. Due to its large precursor ion isolation window, the resulting MS2 are typically multiplexed (several molecules captured in the same spectrum) (Bilbao *et al.*, 2015; Doerr, 2015). The post analysis of these fragmentation spectra requires advanced spectral deconvolution methods. In proteomics applications, large reference spectral libraries can be leveraged to improve the deconvolution process, which has facilitated the adoption of DIA (Gillet *et al.*, 2012; Wang *et al.*, 2019; Zha *et al.*, 2018). However, the application of DIA to metabolomics is more challenging due to lower molecular weights of ions and narrower mass range that increases the multiplexing effect (Zha *et al.*, 2018). While efficient spectral deconvolution methods for metabolomics are also available (Tsugawa *et al.*, 2015), the deconvoluted fragmentation spectra are harder to identify by spectral library matching and by *in silico* annotation because they could be convoluted to some degree or inversely lacked actual fragment ions (Schmid *et al.*, 2020).

To overcome these limitations, we introduce MS2Planner, an Iterative Optimized Data Acquisition (IODA) strategy for maximizing the coverage and quality of MS2 acquisition by untargeted mass spectrometry. While DDA acquisition collects MS2 spectra dynamically, MS2Planner requires a preliminary mass spectrometry experiment performed in MS1 full-scan mode. That preliminary data are used by MS2Planner to compute and schedule the optimal MS2 acquisition path across multiple subsequent iterative experiments. In proteomics, several strategies have been proposed to optimize MS2 coverage (Jaffe *et al.*, 2008; Rudomin *et al.*, 2009; Zerck *et al.*, 2013). MS2Planner is different from them in that it not only maximizes the number of ions with MS2 spectra acquired, but also maintains their quality. Our evaluation showed that MS2Planner outperforms DDA in sensitivity, specificity and quality of MS2 spectra collected. MS2Planner is currently available for Orbitrap-based instruments and could be adapted to other tandem mass spectrometers. Table 2 describes the terminologies used in this article.

**Table 2.** Definitions of the terminologies

Term	Definitions
MS1/MS1 scan	Molecules are ionized and separated by retention time (RT) and mass-to-charge ratio (*m/z*). RT, *m/z* and intensity are obtained for each of the ions.
MS1 intensity	The intensity of the ions. It is acquired during MS1 scan.
MS1 feature	It refers to the whole MS1 profiles from a molecule.
MS1 apex	MS1 scan data are processed by OpenMS workflow. It aligns MS1 features and removes ions found in the background control. It outputs an apex that consists of a tuple made by RT, *m/z*, and intensity of each MS1 feature.
MS1 run	It refers to an entire run of MS1 scans over the RT range of the experiment.
MS2/MS2 scan	Designate fragmentation spectra that results from the fragmentation of molecules detected in MS1 scan and selected for tandem mass spectrometry experiment. It acquires RT, *m/z* and intensity for each ion of the fragments. These data can be used for molecule identification.
Total ion current	It refers to the summation of intensities of ions in a scan or multiple scans.
IODA	Iterative Optimized Data Acquisition: an iterative acquisition strategy that relies on a preliminary analysis for optimizing the acquisition of MS2 scans over one or more iterative targeted-MS2 experiments.
Target ions/inclusion list	It refers to a list of target ions to select for MS2 scan acquisition. When an inclusion list is added to the DDA method, the instrument triggers MS2 scan even if they are not amongst the topN most intense ions observed in the previous MS1 scan. When using the PRM scan, MS2 scans are collected for the target ions regardless of their detection in previous MS1 scans.
Dynamic exclusion	Dynamic exclusion instructs the instrument to stop collecting additional MS2 scan for a period after the first MS2 collection. This avoids collecting duplicate fragment spectra based on *mz* of recently collected ion.
Parallel reaction monitoring	In this acquisition mode, MS2 scan(s) are collected for target ions regardless of their detection in MS1 scan.

## 2 Materials and Methods

### 2.1 Problem formulation

Based on a full preliminary MS1 run, our goal is to schedule an optimal data collection path to maximize the number of distinct MS2 acquisitions while avoiding sub-optimal intensity acquisitions. This problem can be formalized as follows:

(1) $$\underset{X_{t,i}}{\text{argmax}}\sum_{i=1}^{N}1\left(\sum_{t=1}^{T}X_{t,i}>0\right)\;s.t.\;$$

 

(2) $$f\left(\sum_{t=1}^{T}{\text{Int}}_{t,i}*X_{t,i}\right)\geq \text{TIC}$$

 

(3) $$\sum_{t=1}^{T-1}|X_{t+1,i}-X_{t,i}|\leq 2$$

 

(4) $$\sum_{i=1}^{N}X_{t,i}\leq 1$$

 

(5) $$X_{t,i}\in \{0,1\},\;i=1,\ldots ,N,\;\;t=1,\ldots ,T$$

where there are *N* metabolite features and T MS1 scans. ${\text{Int}}_{t,i}$ is the MS1 intensity of metabolite feature *i* at retention *t*, while $X_{t,i}$ is a binary indicator of whether metabolite feature *i* is collected at retention time *t* by MS2. *f* is a function that predicts the total ion current (TIC) of MS2 based on the MS1 intensity. 1 is an indicator function. TIC is a user defined threshold of MS2 TIC.

The objective function (1) maximizes the number of distinct MS2 acquisitions. Constraint (2) means the expected MS2 TIC is greater than the user defined threshold TIC. Constraint (3) guarantees that each metabolite feature is only acquired once within a single interval of retention times. If a feature *i* is collected in two or more separate intervals, e.g. $X_{.,i}=0,0,0,1,1,1,0,0,1,1,0,0$, then $\sum_{t=1}^{T-1}\left|X_{t+1,i}-X_{t,i}\right|=4$, violating the constraint (3). Constraint (4) guarantees that at each retention time, there will be at most one acquisition. Zerck *et al.* also proposed a strategy based on preliminary full MS1 scans, but their optimization criteria does not maximizes the number high-quality features collected (Zerck *et al.*, 2013).

#### Overview of MS2Planner

**Fig. 2.**
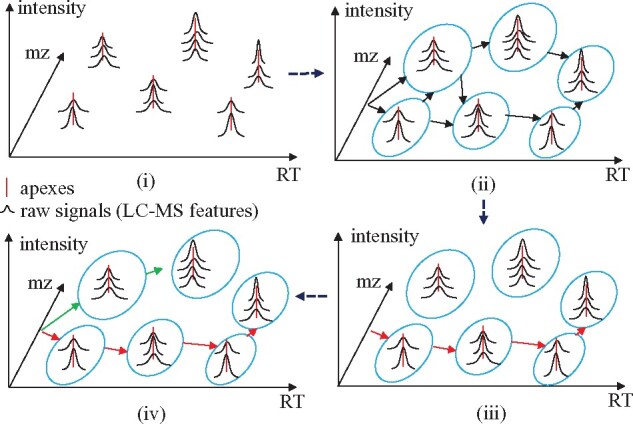
MS2Planner pipeline. The method consists of (i) preprocessing and detecting raw MS1 features, (ii) constructing feature-DAG, (iii) scheduling the optimal path and (iv) excluding the features from this path and iteratively scheduling the next optimal paths

MS2Planner recruits a dynamic programming approach to solve the optimization problem (1). The raw data of the preliminary MS1 run is a list of peaks in triplet format as *m/z*, RT and peak intensity, which are processed by OpenMS to detect MS1 apexes. After preprocessing, MS2Planner algorithm takes both the raw MS1 signals (defined as LC-MS features) and the apexes as input and computes the optimal MS2 acquisition paths in the *m/z*-RT. MS2Planner consists of the following steps: (i) raw MS1 signals are clustered based on their apexes, (ii) a directed acyclic graph (DAG) is constructed on MS1 features, referred to as feature-DAG, (iii) the MS2 acquisition path that maximizes the number of distinct features collected under the sub-optimal intensity constraints in the feature DAG is predicted, (iv) all the features on current path are excluded and the next optimal paths are predicted (Fig. 2). Here, step (i) reduces the possibility of collecting chimeric spectra by targeting at apexes of peaks. The feature-DAG construction algorithm in step (ii) encodes the constraints (2)−(4), while the path finding algorithm in step (iii) guarantees that the paths will cover the maximum number of distinct features. The path scheduled by MS2Planner is the solution of optimization problem (1) (see Supplementary Note 1).

### 2.2 Mass spectrometry feature detection

The preliminary mass spectrometry experiments (acquired in full MS1 scan mode) are processed using OpenMS to detect and align MS1 features (Supplementary Note 2) (Junker *et al.*, 2012; Pfeuffer *et al.*, 2017). MS1 signals are collected on both the biological sample of interest and the background/control sample, and MS1 features are detected using *FeatureFinderMetabo* and aligned across the samples using *FeatureLinkerKD*. Then, the apexes of MS1 features are detected and their intensities in the biological sample and in the background/control sample are reported. Features present in background/control samples are discarded.

### 2.3 Constructing feature-DAG

**Fig. 3.**
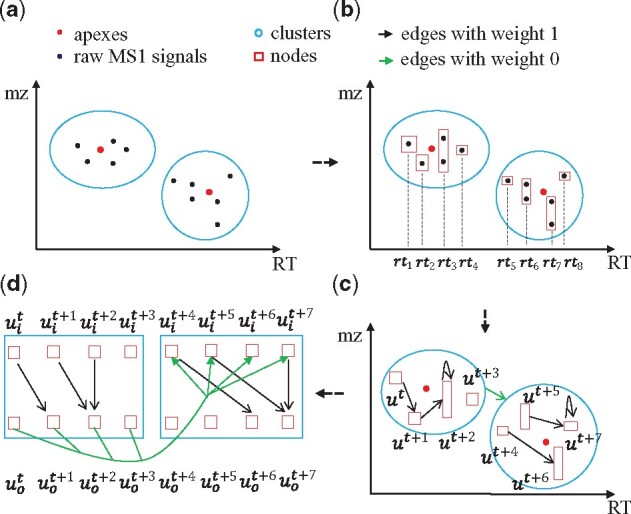
Constructing feature-DAG. Feature-DAG is constructed based on raw MS1 signals in the following steps: (**a**) MS1 signals are clustered based on apexes. (**b**) For each cluster, all raw MS1 signals with the same retention times are regarded as a single node. (**c**) Edges with weight 1 (black) and weight 0 (green) are created depending on retention times and intensity of the nodes. (**d**) Each node is split into an in-node (*u_i_*) and an out-node (*u_o_*) to prevent recollecting the same feature

Feature-DAG is constructed based on raw MS1 features in four steps detailed below (Fig. 3).

#### 2.3.1 Clustering MS1 signals

MS2Planner utilizes raw MS1 signals and apexes from the OpenMS workflow. Raw signals are then mapped to clusters with apexes as centers, using nearest-neighbor model (Fig. 3a) (Altman, 1992).

#### 2.3.2 Creating feature-DAG nodes

Given clusters of MS1 features, MS2Planner bins raw MS1 signals in each cluster based on their retention times (Fig. 3b). The intensity of the node is defined as the sum of intensity of the all raw MS1 signals inside it.

#### 2.3.3 Creating feature-DAG edges

**Fig. 4.**
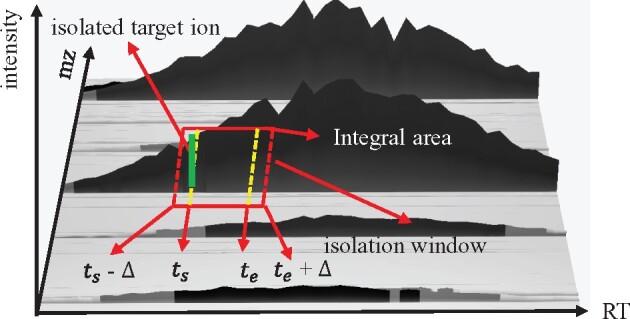
Predicting TIC in *m*/*z-*RT intervals. Total ion current is linearly correlated to the integration of the intensity of all raw MS1 signals in the window. In order to make the prediction more robust against fluctuation in retention times, −Δ and + Δ is added to scan start time (*t*_s_) and scan end time (*t*_e_), respectively, where Δ=0.2 s. Figure is reproduced from Pluskal *et al.* (2010)

First, we connect each node to other nodes in the same cluster with a directed edge of weight 1 if: (i) the retention time of the second node is larger than that of the first node, and (ii) the total ion current (TIC) for the m/z-RT window between the two nodes is higher than a user-defined threshold. MS2Planner trains a linear regression model to predict TIC for each m/z-RT window based on the integral of the intensity of features within the window (Fig. 4 and Supplementary Note 3). In practice, this is equivalent to imposing a threshold on integrated MS1 signal or apex intensity directly. Here, as we assume that the majority of fragmentation spectra detected are not chimeric or multiplexed, TIC can be used as a proxy to estimate signal-to-noise level of a fragmentation mass spectrum (Moore *et al.*, 2000). Hence, constraint (2) is imposed to guarantee that the features collected are high quality.

Second, we connect each node to nodes from other clusters with a directed edge of weight 0 if the retention time of the second node is larger than the first node.

Under different platform/acquisition parameters, the relationship between MS2 TIC and integral of raw MS1 signal could be very different. It is necessary for the user to first run a standard DDA-ex experiment to obtain the training data of a particular platform/acquisition parameters setting, and then learn a customized TIC prediction model with the training data. In this study, the threshold for the TIC of MS2 scan was empirically established based on the performance of the q-Orbitrap instrument used and the target level of sensitivity.

#### 2.3.4 Splitting the nodes

**Fig. 5.**
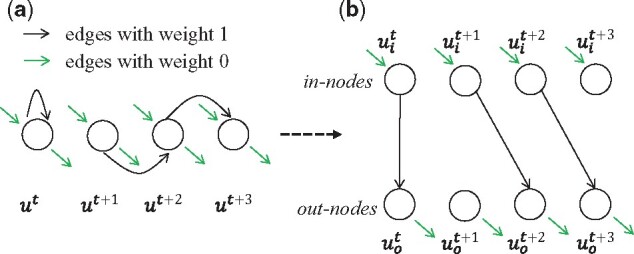
Splitting the nodes. The self-loop edge with weight 1 in node *u^t^* results in duplicate scoring. In order to avoid this, we split each node into in-nodes and out-nodes. Each cluster is entered from an in-node, explored through an edge from an in-node to an out-node, and then exited from an out-node

One of the challenges with finding the maximum score path in feature-DAG is that it is possible for paths to explore and score some clusters multiple times (Fig. 5). In order to avoid this phenomenon, we split each node to an in-node and an out-node. Inside each cluster, all the edges are directed from in-nodes to out-nodes. Between clusters, the edges are directed from out-nodes to in-nodes.

### 2.4 Scheduling the optimal path

MS2Planner schedules the optimal path in m/z/RT plane that maximizes the number of unique features with TIC higher than a threshold (here we use threshold $10^{3}$). This is equivalent to finding the longest path that traverses maximum number of weight 1 edges in the feature-DAG. MS2Planner uses topological sorting to solve this problem efficiently (Kahn, 1962).

### 2.5 Iteratively scheduling the next optimal paths

Once a longest path is found, features in the path are excluded and MS2Planner is applied again to the rest of the features to find the next optimal path. This procedure is continued till reaching a user specified number of iterations.

## 3 Results

### 3.1 Sample preparation

The ‘NIH-NP’ sample consists of a mixture of ground truth molecules from the National Institute of Health natural product library. A biological background of fecal/tomato plant extracts was added to increase the complexity of the sample. The ‘Euphorbia’ plant extract was prepared by extracting the latex of *Euphorbia peplus* with acetonitrile. See the details of sample preparation and mass spectrometry experiments in Supplementary Note 5.

### 3.2 Data collection

The IODA experiments were initiated by analyzing the sample in MS1 full-scan mode. The MS1 data were then processed with the MS2Planner workflow to schedule the optimal path for MS2 acquisition. Subsequently, iterative acquisitions are performed to collect MS2 in targeted mode. Three different methods were used for MS2Planner: DDA with MS2Planner inclusion list (*MS2Planner-DDA-inex*), targeted-MS2 mode with MS2Planner inclusion list (*MS2Planner-Targeted-inex*) or targeted-MS2 mode with MS2Planner inclusion list without dynamic exclusion (*MS2Planner-Targeted-in*). Five replicates of standard DDA (DDA-ex) were collected as baseline comparison. Table 3 details various features of these approaches.

**Table 3.** Comparison of acquisition methods used in the study

Method	MS2 acquisition mode	DDA	Inclusion list	Dynamic exclusion	Acquire MS2 from MS1
MS1 full scan	None	No	No	No	N/A
DDA-ex	DDA	Yes	No	Yes	Yes
MS2Planner-DDA-inex	DDA + targeted MS2	Yes	Yes	Yes	Yes
MS2Planner-Targeted-inex	Targeted MS2	No	Yes	Yes	Yes
MS2Planner-Targeted-in	Targeted MS2	No	Yes	No	No

***Note*:** In standard DDA (*DDA-ex*), acquisition is done in DDA mode (top five ions detected in MS1 scans), and MS2 dynamic exclusion is used to avoid collecting duplicate fragment spectra on recently collected ions. The *MS2Planner-DDA-inex* is a hybrid method that uses DDA for the ions detected in the previous MS1 survey scans, but prioritizes the acquisition of MS2 spectra for target ions which are predicted by *MS2Planner*. In the *MS2Planner-Targeted-inex* mode, the MS2 are collected only once for the targets in the inclusion list produced by MS2Planner. In the *MS2Planner-Targeted-in* method, the MS2 scans are collected for the targets regardless of their detection in the previous MS1 scans and no dynamic exclusion is used which can result in the acquisition of additional MS2 scans for target ion if extra duty cycle is available.

### 3.3 Ms2planner detects more high quality unique compounds

**Fig. 6.**
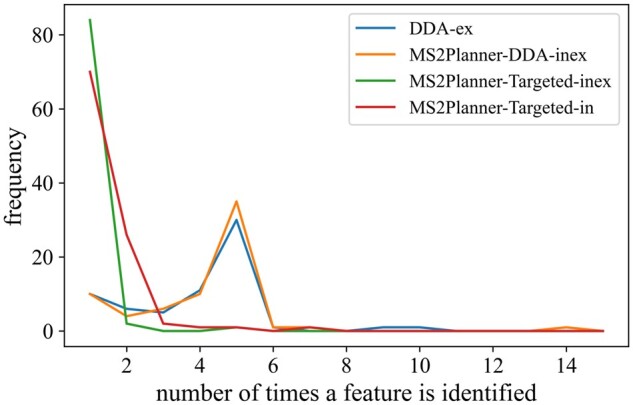
Distribution of frequency of duplicate feature collection. In the MS2Planner IODA experiments, 98.8% and 95.1% of features are collected 1–2 times for *MS2Planner-Targeted-inex* and *MS2Planner-Targeted-in*, respectively, indicating a minimal redundancy in fragment spectra acquisition. In *DDA-ex* and *MS2Planner-DDA-inex*, this rates is approximately 25% indicating that 75% are redundantly collected more than 2 times as these strategies collect MS2 for the most intense features detected (once in each acquisition)

It is known that *DDA-ex* is biased toward collecting MS2 for the most intense ions. When running technical replicates in DDA, the instrument recollects MS2 for duplicate most intense ions. Figure 6 illustrate that 95.1% of the features in MS2Planner-Targeted-in mode appear in 1–2 MS2 spectra, while it is only 24.6% in *DDA-ex* mode. Therefore, as expected, *MS2Planner-Targeted-in* has a lower redundancy in fragment spectra acquisition.

**Fig. 7.**
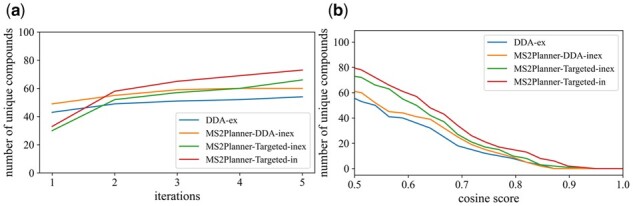
Benchmarking on number of unique compounds and spectrum quality. (**a**) Number of unique compounds versus number of runs for different methods. *MS2Planner-Targeted-in* collects most unique compounds over five iterations among all methods. (**b**) Number of unique compounds identified at different cosine thresholds. *MS2Planner-Targeted-in* produce more high quality spectra than other methods

*MS2Planner-Targeted-in* also outperforms other methods in the cumulative number of detected unique ground truth molecules (Fig. 7a). Only spectral annotations corresponding to ‘ground truth’ molecules that are known to be present in the NIH-NP sample are considered. These annotations are required to have: a cosine score ≥0.5, shared peaks ≥2 and part-per million (ppm) error ≤20 compare to reference spectra. Over five experiments, with the same filter parameters, *MS2Planner-Targeted-in, MS2Planner-Targeted-inex and MS2Planner-DDA-inex allowed to identify +38.6% (79) and +28.1% more ground truth molecules compared to DDA-ex (57), while MS2Planner-DDA-inex identified +8.7% more (57). Furthermore, we used the* modified cosine score between the experimental MS2 spectra and the reference library spectra as a proxy to estimate the quality of experimental spectra collected. The cosine score represents the similarity of two MS2 spectra and ranges from 0 (not similar at all) to 1 (identical). Figure 7 shows that *MS2Planner-Targeted-in* produces also more high quality unique compounds than other three methods for all cosine thresholds.

#### MS2Planner improves sensitivity and specificity of data acquisition

**Fig. 8.**
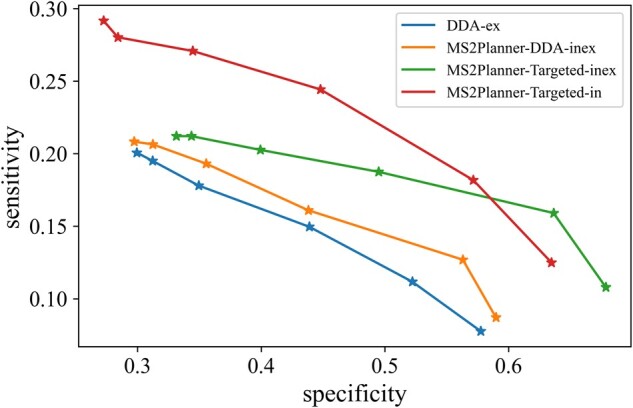
Sensitivity and specificity of different acquisition methods. Sensitivity and specificity for the NIH-NP dataset. These are computed from the annotation rate obtained by spectral library search against reference spectra. Here for cosine thresholds ranging from 0.1 to 0.6. *MS2Planner-Targeted-in* and *MS2Planner-Targeted-inex* outperform *DDA-ex* and *MS2Planner-DDA-inex*

Sensitivity and specificity are computed from the ground truth compounds annotated from spectral library search. Sensitivity (true positive rate) is denoted as the number unique MS2 annotated as ground truth molecules over the number ground truth molecules in the sample. Specificity is defined as the number of unique MS2 ground truth molecules that were identified over the unique number of fragment spectra collected. Figure 8 shows that *MS2Planner-Targeted-in* and *MS2Planner-Targeted-inex* outperform *DDA-ex* and *MS2Planner-DDA-inex*. At cosine threshold of 0.5, the sensitivity of *DDA-ex*, *MS2Planner-Targeted-in*, *MS2Planner-DDA-inex* and *MS2Planner-Targeted-inex* is 11.2%, 18.2%, 12.7% and 15.9% respectively, while their specificity is 52.2%, 57.1%, 56.3% and 63.6%.

### 3.4 Performance on the *Euphorbia* plant sample

**Fig. 9.**
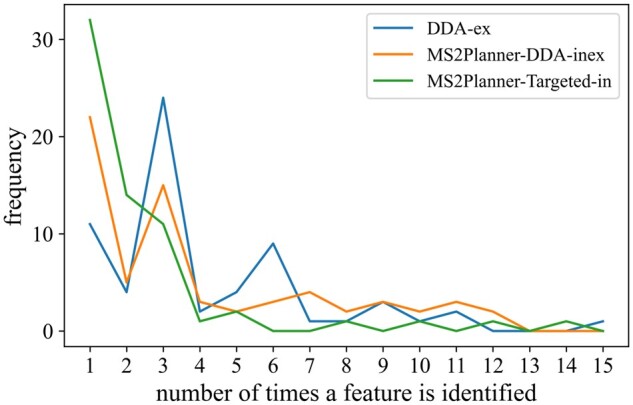
Distribution of frequency of duplicate feature collection on the *Euphorbia* plant data. DDA-ex, MS2Planner-DDA-inex and MS2Planner-Targeted-in collected 23.8%, 40.9%, 71.9%, of MS1 features 1–2 times

To benchmark MS2Planner on a more complex plant extract, we performed a similar analysis on a *Euphorbia* extract. Mass spectral data were searched against all public spectral libraries and NIST. Over three replicates *DDA-ex* and three iterative acquisitions were performed for *MS2Planner-Targeted-in*, *MS2Planner-DDA-inex* collected 23.8%, 71.9%, 40.9% of features only 1–2 times. *MS2Planner-Targeted-in* has a lower redundancy in fragment spectra acquisition (Fig. 9).

## 4 Discussion

Standard DDA and its variants use a greedy strategy that always collect the most abundant ions for MS2 acquisition based on the previous MS1 scan. MS2Planner, on the contrary, first acquires a complete MS1 profile of a sample, and then schedules the path in the m/z/RT plane to maximize the coverage and the quality of MS2 acquired. MS2Planner is an IODA strategy for untargeted mass spectrometry analysis who has ‘see the future’ and can plan ahead. Our evaluation showed that IODA with MS2Planner offers higher annotation rate than standard DDA, as well as higher sensitivity and specificity. While its iterative nature implies more experimental time, IODA with MS2Planner expands the analytical depth of untargeted mass spectrometry. In addition, and unlike DIA, the supplementary MS2 spectra collected can be readily employed for *in silico* annotation. We argue that the possibility of high coverage in qualitative MS2 coupled with the recent methodological advances in MS2 annotation is opening the ‘deep metabolomics’ era, where metabolites content can be detected and annotated at unprecedented depths. MS2Planner can be applied on other mass spectrometry platforms but is particularly adapted to q-Orbitrap instrument as it empowers the dynamic C-trap filling capability. Indeed, ion accumulation time is adjusted for each MS2 based on the input stream of ions available. Actually, the modulation of C-trap specific parameters (maximum filling time and maximum number of ions accumulated) has the potential to further increase the sensitivity of IODA with MS2Planner. From a practical standpoint, a typical use case for deep metabolomics acquisition would be analyzing all samples by high-throughput DDA or DIA and performing IODA on representative sample(s) for comprehensive MS2 acquisition and annotation.

While MS2Planner improves on the sensitivity of the existing methods, currently the sensitivity of all the presented methods remain below 30%. Various experimental and computational improvements are needed to achieve higher sensitivity rates, including improvements in retention of small molecules in liquid chromatography, improvements in acquisition of low intensity features, and improvement in spectral library search. In practice, to reach sensitivity rates near 100%, one needs to conduct ionization in both positive and negative modes (as many small molecules do not fragment in positive mode), while using both liquid and gas chromatography with different chromatographic conditions.

## Supplementary Material

btab279_Supplementary_Data
