# Supplementary material for: MS2Planner: improved fragmentation spectra coverage in untargeted mass spectrometry by iterative optimized data acquisition
Source: Bioinformatics. 2021 Jul 12;37(Suppl 1):i231–6. doi: 10.1093/bioinformatics/btab279 (PMC8336448; doi:10.1093/bioinformatics/btab279)
Supplement: btab279_Supplementary_Data [file btab279_supplementary_data.pdf]

Supplementary Note 1: MS2Planner provides a solution of the optimization problem.

Lemma. *The path reported by MS2Planner can be converted into a valid solution of the optimization problem (1).*

Suppose MS2Planner gives a path of $2P+2$ nodes covering P metabolite feature clusters (see subsection "Constructing feature-DAG" of Method section) as follows:

$$source, u_{m_1}^{t_{m_1,i}}, u_{m_1}^{t_{m_1,o}}, u_{m_2}^{t_{m_2,i}}, u_{m_2}^{t_{m_2,o}}, \cdots, u_{m_P}^{t_{m_P,i}}, u_{m_P}^{t_{m_P,o}}, sink$$

where $u_{m_p}^{t_{m_p,i}}$ and $u_{m_p}^{t_{m_p,o}}$ stand for the in-node and the out-node of the cluster of metabolite feature m_p. $t_{m_p,i}$ and $t_{m_p,o}$ are the corresponding retention times.

The retention times of the path are in ascending order

$$t_{m_1,i} \leq t_{m_1,o} < t_{m_2,i} \leq t_{m_2,o} < \cdots < t_{m_P,i} \leq t_{m_P,o}$$

Based on this path, we construct a solution for the optimization problem as follows:

$$X_{t,m_p} = \begin{cases} 1 & t_{m_p,i} \leq t \leq t_{m_p,o} \\ 0 & \text{otherwise} \end{cases}$$

$$m_p \in \{m_1, m_2, ..., m_P\}$$

We will prove that this solution satisfies constraints (2)-(5).

For constraint (2), according to subsection "creating feature-DAG edges", there will be an edge between the in-node $u_{m_p}^{t_{m_p,i}}$ and and out-node $u_{m_p}^{t_{m_p,o}}$ of metabolite feature m_p if and only if

$$TIC \leq f(\sum_{t=t_{m_p,i}}^{t_{m_p,o}} Int_{t,m_p})$$

Then we can obtain

$$\begin{aligned}
TIC \leq & f(\sum_{t=t_{m_p,i}}^{t_{m_p,o}} Int_{t,m_p}) \\
= & f(\sum_{t=t_{m_p,i}}^{t_{m_p,o}} Int_{t,m_p} * 1) \\
= & f(\sum_{0 \leq t < t_{m_p,o}} Int_{t,m_p} * 0 + w \sum_{t=t_{m_p,i}}^{t_{m_p,o}} Int_{t,m_p} * 1 + \sum_{t_{m_p,o} < t \leq T} Int_{t,m_p} * 0) \\
= & f(\sum_{0 \leq t < t_{m_p,o}} Int_{t,m_p} * X_{t,m_p} + \sum_{t=t_{m_p,i}}^{t_{m_p,o}} Int_{t,m_p} * X_{t,m_p} + \sum_{t_{m_p,o} < t \leq T} Int_{t,m_p} * X_{t,m_p}) \\
= & f(\sum_{t=0}^{T} Int_{t,m_p} * X_{t,m_p})
\end{aligned}$$

Therefore, constraint (2) holds.

For constraint (3), MS2Planner never visit the same feature cluster it has visited before, therefore constraint (3) is satisfied.

For constraint (4), suppose it is violated. Then at some retention time t, at least two metabolite feature clusters m_{p_1} and m_{p_2} are visited (suppose $t_{m_{p_1},i} < t_{m_{p_2},i}$). Therefore,

$$\begin{aligned} t_{m_{p_1},i} \leq & t \leq t_{m_{p_1},o} \\ t_{m_{p_2},i} \leq & t \leq t_{m_{p_2},o} \end{aligned}$$

which contradicts with the fact that

$$t_{m_{p_1},i} \leq t_{m_{p_1},o} < t_{m_{p_2},i} \leq t_{m_{p_2},o}$$

Therefore, the assumption that constraint (4) is violated is incorrect. Constraint (4) is satisfied.

Constraint (5) is satisfied since X_{t,m_p} is either 0 or 1.

Therefore, MS2Planner gives a valid solution for the optimization problem. ■

Theorem. *MS2Planner provides an optimal solution to the optimization problem (1).*

Assume MS2Planner gives a path of $2P + 2$ nodes covering P metabolite feature clusters as is defined in *Lemma*, which by definition is the longest path of the feature-DAG. We prove that there is no path that can cover more than P metabolite feature clusters for the optimization problem.

Suppose there is a path that can cover $P + 1$ metabolite features in the optimization problem:

$$X_{t,m_p} = \begin{cases} 1 & t_{m_p,i} \leq t \leq t_{m_p,o} \\ 0 & \text{otherwise} \end{cases}$$

$$m_p \in \{m_1, m_2, ..., m_{P+1}\}$$

where $t_{m_p,i}$ and $t_{m_p,o}$ are the starting and ending retention time of metabolite feature m_p, and they are in the ascending order

$$t_{m_1,i} \leq t_{m_1,o} < t_{m_2,i} \leq t_{m_2,o} < \cdots < t_{m_{P+1},i} \leq t_{m_{P+1},o}$$

According to constraint (2)-(5), there should be an edge with weight 1 between $u_{m_p}^{t_{m_p,i}}$ and $u_{m_p}^{t_{m_p,o}}$, $m_p \in \{m_1, m_2, ..., m_{P+1}\}$ in the DAG. Since there are $P + 1$ features, there should be $P + 1$ such edges in total. According to the definition of the DAG, there will also be an edge with weight 0 between $u_{m_p}^{t_{m_p},o}$ and $u_{m_{p+1}}^{t_{m_{p+1}},i}$.

By further adding the edge of 0 weight between $u_{m_1}^{t_{m_1},i}$ and source node, as well as the edge of 0 weight between $u_{m_{P+1}}^{t_{m_{P+1}},o}$ and sink node, we can obtain the final path of the feature-DAG, whose total weight is P+1. This is contradicting with the fact that the length of longest path of feature-DAG is P. Therefore, there is no solution that can cover more than P metabolite features in the optimization problem. The solution given by MS2Planner is the optimal. ■

Supplementary Note 2: OpenMS workflow. Mass spectrometry data (.mzML) is preprocessed by feature detection and alignment module (e.g. FeatureFinderMetabo). It generates a table of features with mass to charge, retention time, charge and intensity. Figure S1 shows a example workflow to preprocess MS1 .mzML file into .mzTab file. Sample and control .mzML are inputs and it produces a merged .mzTab file.

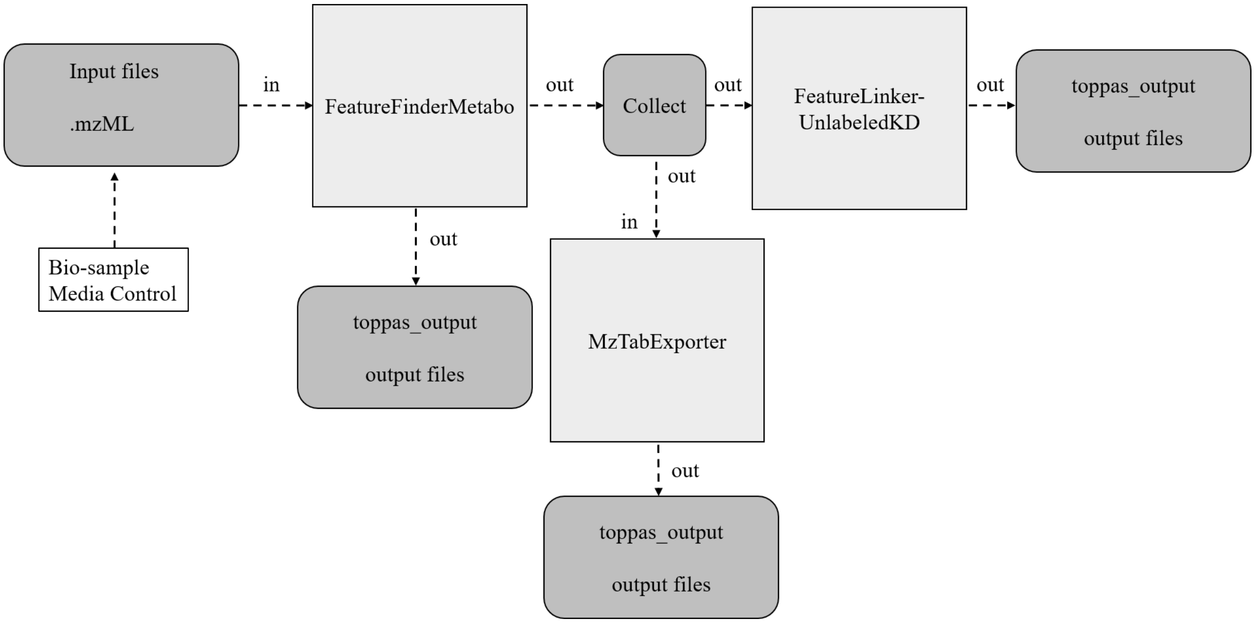

Fig S1. OpenMS preprocessing workflow. The workflow takes biological sample and media control MS1 as input and processes MS1 data with FeatureFinderMetabo module. It outputs .mzTab file of detected features.

Supplementary Note 3: Predicting TIC. Given MS1 scans, MS2Planner estimates the expected TIC for a MS2 acquisition based on either (i) the integral of the intensity of all raw MS1 signals at a m/z and RT window (Figure S2a) or (ii) the apex intensity (Figure S2b). Under the log scale, the Pearson correlation between the integral of the raw MS1 signal and MS2 TIC is 0.78, while the Pearson correlation between apexes intensity and TIC is 0.36, indicating that integral of raw MS1 signals is a better predictor of TIC.

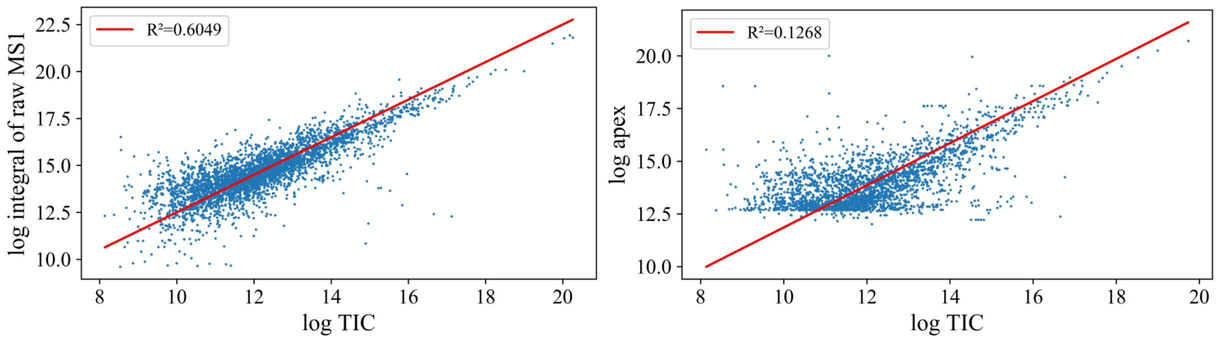

(a) (b)

Fig S2. Predicting TIC from MS1 signals. (a) Log of TIC in MS2 versus log integral of MS1 signals (Pearson correlation of 0.78). (b) Log of TIC in MS2 versus log of MS1 apexes (Pearson correlation of 0.36). This demonstrates that integration of raw MS1 signal predicts TIC better than apexes.

As MS1 retention times might be slightly off from MS2 retention times (Figure S3), in practice,

MS2Planner relaxes the start and end of the integral window by $\Delta = 0.2$ sec (Figure 4).

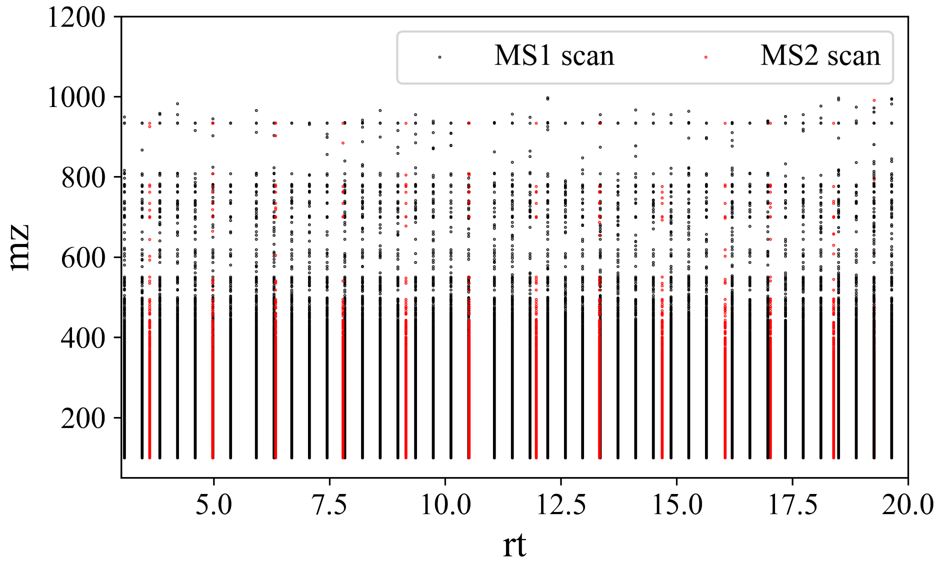

Fig S3. MS1 and MS2 scan retention time shifts. Retention time shifts between MS1 and MS2 scan are observed. While some MS1 and MS2 scans overlap, some have small shifts in retention time.

Supplementary Note 4: Path visualization. We show that the sampling paths of DDA-ex replicates (Figure S4a) overlap with each other more often than the sampling trajectories of MS2Planner-Targeted-in (Figure S4b). In addition,

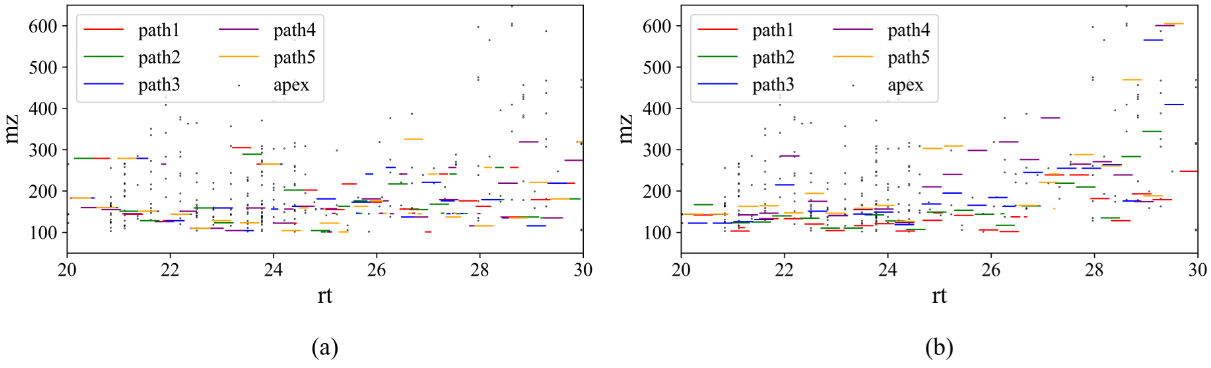

Fig S4. Path visualization for DDA-ex and MS2Planner-Targeted-in. The top five paths in m/z/RT plane for (a) DDA-ex and (b) MS2Planner-Targeted-in.

Supplementary Note 5: Sample preparation and data collection.

Mass Spectrometry Analysis of the NIH-NP dataset

Sample prepration A mixture of 341 natural products (NP) from the "NIH NPAC ACONN" collection was prepared and diluted into acetonitrile/methanol/water (2/2/1) at approximative concentration of 0.1uM [19] Prior to the analysis, representative biological extracts (human fecal and tomato seedling extracts) were added to the natural product mixture to complexify the sample [32]. The biological extracts added to the mixture represented 1/3 of total volume of the final sample used for the analyses.

LC-MS general Liquid chromatography and mass spectrometry experiments. Samples were analyzed using ultra high performance liquid chromatography (Vanquish, Thermo Scientific) coupled to a quadrupole-Orbitrap (q-Orbitrap) mass spectrometer (Q Exactive, Thermo Scientific). The q-Orbitrap was fitted with an electrospray source (HESI-II) operating in positive ionisation mode. The source used the following parameters: spray voltage, +3500 V; heater temperature, 380°C; capillary temperature, 320°C; S-lens RF, 60 (arb. units); sheath gas flow rate, 50 (arb. units); and auxiliary gas flow rate, 15 (arb. units), sweep gas flow rate, 3 (arb. units). All solvents and reagents were LC-MS grade.

Liquid chromatography The chromatographic column was a Phenomenex Kinetex C18 1.7 µm (100A) 100 x 2.1 mm equipped with guard cartridges (Phenomenex). The mobile phases consisted of A (100% water + 0.1% formic acid) and B (100% acetonitrile + 0.1% formic acid), and the flow rate was set to 500 µL/min throughout the experiment, and the column maintained at 40℃. The main chromatographic elution method was set to: 0.00 - 0.15 min, isocratic 25% B; 0.15 - 8.20 min, from 25% to 100% B; 8.20 - 8.60 min, isocratic 100% B; 8.60 - 8.70 min, from 100% to 25% B; 8.70 - 10.00 min, isocratic 25% B. The 50 minutes long chromatographic elution method was set to: 0.00 - 0.75 min, isocratic 25% B; 0.75 - 41.00 min, from 25% to 100% B; 41.00 - 43.00 min, isocratic 100% B; 43.00 - 43.50 min, from 100 to 25% B; 43.50 - 50.00 min, isocratic 25% B.

Mass Spectrometry MS1: polarity, positive; in-source CID, 0 (eV); microscans, 1; resolution, 35K; AGC target, 5e6; maximum injection time, 200 (ms); number of scan, 1; scan range, 100 to 1000 *m/z*, spectrum data: profile.

Standard DDA (DDA-ex): Global settings - Chromatographic peak width, 16 (sec.), ion tolerance, 5 (mmu), General parameters - polarity, positive; in-source CID, 0 (eV); default charge, 1, inclusion, off; exclusion, off - Full MS parameters - microscans, 1; resolution 17.5K, AGC target, 1e6; maximum injection time, 100 (ms), number of scans, 1; scan range, 100 to 1000 *m/z*, spectrum data: profile. dd-MS2 parameters - microscans, 1, resolution, 17.5K, AGC target, 5e5; maximum injection time, 350 ms; TopN, 3 (loop count, 3; MSX count, 1); isolation window 1.5 *m/z*; isolation offset: 0.0 *m/z*; fixed first mass 50.0 *m/z*; stepped normalized collision energies, 20, 30, 40 (eV); spectrum type, centroid. dd settings - minimum AGC target, 3.6e4 (intensity threshold, 1e5); apex trigger, 2 to 8 (sec.); charge exclusion, none; peptide match, n/a; exclude isotope, on; dynamic exclusion, 15 (sec.); if idle, n/a.

MS2Planner-DDA-inex: The standard DDA method was used with the following changes in parameters: General parameters - inclusion, on. dd settings - if idle, pick others. An inclusion list for target ions predicted by MS2Planner was specified for each iterative IODA experiment.

MS2Planner-Targeted-inex: The standard DDA method was used with the following changes in parameters: General parameters - inclusion, on. dd settings - if idle, do not pick others. An inclusion

list for target ions predicted by MS2Planner was specified for each iterative IODA experiment.

MS2Planner-Targeted-in: The method consists of one Full MS cycle and one PRM cycle. The Full MS scan uses the same parameters as in the DDA experiments. The PRM scan uses the following parameters: Global settings and General parameters, similar to the DDA experiment, except for inclusion: on. MS2 parameters were similar to DDA experiment - microscans, 1; resolution 17.5K, AGC target, 5e5; maximum injection time, 350 (ms); Loop count, 5; MSX count, 1; MSX isochronous, on, isolation window, 1.5 (m/z), stepped normalized collision energies, 20, 30, 40 (eV). An inclusion list for target ions predicted by MS2Planner was specified for each iterative IODA experiment.

Mass Spectrometry Analysis of the Euphorbia plant

Sample preparation The latex of *Euphorbia peplus* was collected from several specimen found in La Jolla California (May 2020). The botanical identification was carried out by LFN and a voucher specimen was kept at Skaggs School of Pharmacy at UCSD. For the collection, incisions were made on the aerial parts and the exudating latex drops (approximatively 100 drops) were harvested in 2 mL of acetonitrile (LC-MS grade). The solution was centrifugated at 5000 rpm for 15 minutes and the supernatant was resuspended for LC-MS analysis. Note that full protective equipment is needed for the collection and preparation of Euphorbia plants.

Liquid chromatography The chromatographic column was a Phenomenex Kinetex C18 1.7 µm (100A) 50 x 2.1 mm equipped with guard cartridges (Phenomenex). The mobile phases consisted of A (100% water + 0.1% formic acid) and B (100% acetonitrile + 0.1% formic acid), and the flow rate was set to 500 µL/min throughout the experiment, and the column maintained at 40℃. The chromatographic elution method was set to: 0.00 - 0.15 min, isocratic 40% B; 0.15 - 4.50 min, 40% to 65% B; 4.50 - 5.70 min, 65% to 100% B; 5.70 - 6.10 min, isocratic 100% B; 6.10 - 6.20 min, 100% to 40% B, - 6.2- 7.00 min, isocratic 40% B. The 21 minutes long chromatographic elution method was set to: 0.00 - 0.45 min, isocratic 40% B; 0.45 - 13.50 min, 40% to 65% B; 13.50 - 17.10 min, 65% to 100% B; 17.10 - 18.30 min, isocratic 100% B; 18.30 - 18.60 min, 100% to 40% B, 18.60 - 21.00 min, isocratic 40% B. The 35 minutes long chromatographic elution method was set to: 0.00 - 0.75 min, isocratic 40% B; 0.75 - 22.50 min, 40% to 65% B; 22.50 - 28.50 min, 65% to 100% B; 28.50 - 30.50 min, isocratic 100% B; 30.50 - 31.00 min, 100% to 40% B, 31.00 - 35.00 min, isocratic 40% B.

Mass spectrometry MS1: polarity, positive; in-source CID, 0 (eV); microscans, 1; resolution, 35K; AGC target, 1e6; maximum injection time, 200 (ms); number of scan, 1; scan range, 100 to 1000 m/z, spectrum data: profile.

Standard DDA experiment (DDA-ex) : The parameters used were similar to above except for: Global settings - Chromatographic peak width, 15 (sec.) - dd-MS2 parameters - AGC target, 5e5; stepped normalized collision energies, 30 (eV); spectrum type, centroid. dd settings - apex trigger, 2 to 7(sec.); dynamic exclusion, 12 (sec.).

For the DDA experiment with inclusion list (MS2Planner-DDA-inex) , the standard DDA method was used with the following changes in parameters: General parameters - inclusion, on. dd settings

- if idle, pick others. An inclusion list for target ions predicted by MS2Planner was specified for each iterative IODA experiment.

For the targeted-MS2 experiments (MS2Planner-Targeted-in) : the method consists of one Full MS cycle and one PRM cycle. The Full MS scan uses the same parameters as in the DDA experiments. The PRM scan uses the following parameters: Global settings and General parameters, similar to the DDA experiment, except for inclusion: on. MS2 parameters were similar to DDA experiment - microscans, 1; resolution 17.5K, AGC target, 5e5; maximum injection time, 450 (ms); Loop count, 5; MSX count, 1; MSX isochronous, on, isolation window, 1.5 (m/z), stepped normalized collision energies, 30 (eV).An inclusion list for target ions predicted by MS2Planner was specified for each iterative IODA experiment.

Mass Spectrometry Data analysis The mass spectrometry data were analysed by classical molecular networking running on the GNPS web-platform (https://gnps.ucsd.edu). The MS2 spectra were first clustered and searched against spectral libraries available at GNPS. For the NIH-NP dataset, only spectral matches from the corresponding spectral library (NIH Natural Product library) were considered. The job task ID are indicated S1 in Supplementary Notes 6.

Supplementary Note 6: GNPS molecular networking jobs.

Dataset	Method	GNPS job ID
NIH-NP	DDA-ex	a2c47c1de2574a5cb81ad0c27b792008
NIH-NP	MS2Planner-DDA-inex	4067f9de2fe04c07a236987b6a03d224
NIH-NP	MS2Planner-Targeted-inex	ad0098ba424145488876f76cfc3a894d
NIH-NP	MS2Planner-Targeted-in	079ecc86ca1f49bda3251055ac9ae314
Euphorbia plant	DDA-ex	77743083252a4a5b98f255b6b18ece2c
Euphorbia plant	MS2Planner-DDA-inex	39cb3c3b0db44887803035b1ecfd645e
Euphorbia plant	MS2Planner-Targeted-in	c301b9cbe7284fb6b6dec3e2e1d7697e

Table S1: GNPS job descriptions and job ID for data used in the paper.
